# Prescribing of long-term antibiotics to adolescents in primary care: a retrospective cohort study

**DOI:** 10.3399/BJGP.2021.0332

**Published:** 2021-10-05

**Authors:** Mark Lown, Sam McKeown, Beth Stuart, Nick Francis, Miriam Santer, George Lewith, Fangzhong Su, Michael Moore, Paul Little

**Affiliations:** University of Southampton, Southampton.; University of Southampton, Southampton.; University of Southampton, Southampton.; University of Southampton, Southampton.; University of Southampton, Southampton.; University of Southampton, Southampton.; University of Southampton, Southampton.; University of Southampton, Southampton.; University of Southampton, Southampton.

**Keywords:** antibiotic prescriptions, antibiotics, general practice, antimicrobial drug resistance

## Abstract

**Background:**

Antibiotic overuse is linked to increased risk of antimicrobial resistance. Long-term antibiotics are commonly used for treating acne and prophylaxis of urinary tract infection. Their contribution to the overall burden of antibiotic use is relatively unknown.

**Aim:**

To describe the volume of commonly prescribed long-term (≥28 days) antibiotic prescriptions in adolescents and young adults, trends over time, and comparisons with acute prescriptions.

**Design and setting:**

A retrospective cohort study using UK electronic primary care records.

**Method:**

Patients born between 1979 and 1996 and with data in the Care and Health Information Analytics database were included. The main outcome measures were antibiotic prescription rates per 1000 person-years and antibiotic prescription days per person-year between the ages of 11 and 21.

**Results:**

In total, 320 722 participants received 710 803 antibiotic prescriptions between the ages of 11 and 21 years from 1998 to 2017. Of these 710 803 prescriptions, 191 443 (26.93%) were for long-term antibiotics (≥28 days and ≤6 months in duration). Long-term antibiotics accounted for more than two-thirds (72.48%) of total antibiotic exposure (days per person-year). Total long-term antibiotic prescribing peaked in 2013 at just under 6 days per person-year and declined to around 4 days in 2017.

**Conclusion:**

Among adolescents and young adults, exposure to long-term antibiotics (primarily lymecycline used for acne) was much greater than for acute antibiotics and is likely to make an important contribution to antimicrobial resistance. Urgent action is needed to reduce unnecessary exposure to long-term antibiotics in this group. Increasing the use of, and adherence to, effective non-antibiotic treatments for acne is key to achieving this.

## INTRODUCTION

Antimicrobial resistance (AMR) is a major threat to global health as new resistance mechanisms emerge and spread globally.^1^ A growing number of common infections are becoming harder to treat because of AMR, which can lead to longer hospital stays, higher medical costs, and increased mortality.^1^ Unnecessary and inappropriate use of antibiotics promotes the emergence and spread of resistant bacteria.^2^ This effect not only increases the population-level carriage of organisms resistant to first-line antibiotics, but can also increase the use of second-line antibiotics in the community.^3^ Up to half of all antibiotic usage is thought to be inappropriate.^4^

Increased consumption of antibiotics may not only produce greater resistance at the individual patient level but may also lead to greater resistance at the community, country, and regional levels.^5^ Population-level antibiotic pressure may have more effect on an individual risk for resistant organisms than individual antimicrobial usage.^6^ Exposure to an antibiotic not only induces resistance to that antibiotic, but can also induce resistance to other antibiotic classes.^7^ Cross-resistance can occur via various mechanisms, such as co-selection, collateral resistance, collateral sensitivity, or by simply killing competing bacteria.^8^

Repeat use of antibiotics has a stronger association with antibiotic resistance than first use.^8^ Both the dosage and duration of antibiotic therapy may have effects on resistance, with lower doses and longer durations being linked with increased risk of AMR.^7^ Reversal of resistance is complex and resistance might persist for many years despite the introduction of antimicrobial containment and stewardship programmes.^7^ Prudent antibiotic prescribing has been identified as an important strategy to curb AMR including avoiding unnecessary prescriptions, delaying prescriptions when possible, favouring narrow-spectrum over broad-spectrum antibiotics, and optimising treatment duration.^9^^–^10

Long-term antibiotics are commonly used for the treatment of acne^11^^–^12 and prophylaxis of urinary tract infection,^13^ and population exposure to long courses of low-dose antibiotics are associated with increased risk of AMR. Treatment of acne with antibiotics is associated with an increased risk of common infections.^14^^–^15 Acne affects over 90% of teenagers.^11^ There are approximately eight consultations per 100 person-years for acne in 12- to 18-yearolds in the UK and oral antibiotics are the most common acne-related medication prescribed.^16^ Dermatologists prescribe more oral antibiotic courses per clinician than any other specialty, and many of these courses of antibiotics are prescribed for several months in duration.^17^ Non-antibiotic treatments, particularly topical treatments, are more appropriate for the majority of acne.^12^

**Table:** How this fits in

Previous work investigating antibiotic prescribing in primary care has focused primarily on acute antibiotic prescribing, with an emphasis on antibiotic choice and the number of prescriptions issued. This study provides estimates of the overall burden of long-term antibiotic prescriptions in adolescents. This is higher than previously thought, and contributes much more than short-term antibiotics to the number of days of antibiotics used, and is therefore likely to contribute significantly more to the promotion of antibiotic resistance. The use of long-term antibiotics (primarily tetracyclines for acne) contributes significantly to this burden. Non-antibiotic topical treatments are as effective as oral antibiotics for most people with acne, and steps to promote the effective use of these treatments may lead to reductions in antimicrobial resistance without impairing acne outcomes.

Previous work investigating antibiotic prescribing in primary care has focused on antibiotic choice^9^ and the number of prescriptions issued rather than the number of units or prescription days. Describing antibiotic prescriptions linked to specific conditions or indications may underestimate total prescribing.^9^ Other work has focused on the identification of inappropriate antibiotic use but did not consider long-term antibiotic prescriptions, the contribution of which to the overall burden of antibiotic use is relatively unknown.^10^ This study therefore determined the rates and trends, and overall burden, of long-term antibiotic prescriptions in an adolescent cohort in primary care in Hampshire, UK.

## METHOD

### Design and setting

A retrospective longitudinal population-based cohort study was conducted using data from the Care and Health Information Analytics database (CHIA) for the period January 1998 to August 2017. The CHIA database is an anonymised analytical database with data from around 130 general practices across Hampshire containing information on over 1.24 million residents.^18^ Data were accessed via the CHIA governance team, and programming and extraction carried out by a member of the CHIA team. Extracted data included all antibiotic prescriptions. Data were not restricted based on consultations or Read codes (a coded thesaurus of clinical terms used in the NHS). Analyses were performed by year and denominators were calculated using all participants registered with a practice, excluding those who were deceased or had moved out of the area. Only oral antibiotic prescriptions for tablets/ capsules were included in the analysis.

CHIA was used to extract prescription data for patients born between 1979 and 1996 who attended one of the included GP surgeries at least once during adolescence. Antibiotic classes and specific antibiotics were selected using British National Formulary (BNF, which provides key information on the selection, prescribing, dispensing, and administration of medicines in the UK) codes, and both generic and brand names of antibiotics were included in the analysis for the antibiotics listed in Supplementary Table S1. Long-term antibiotic prescriptions were defined as prescriptions for 28 days’ or more supply of antibiotics. This definition has been applied elsewhere for acne prescriptions.^15^ The quantity of medication prescribed for each antibiotic was identified. Antibiotic prescriptions where the quantity prescribed was inferred (by dosing regimens) to be greater than 6 months were excluded as these were thought likely to be errors (issuing prescriptions for longer durations is not recommended). In England, for medicines commonly prescribed for long-term conditions, 93% of the total volume in 2019 was for 3 months or less. Long-term antibiotics were selected based on clinical indications listed in the BNF for acne and infection prophylaxis, and some key short-term antibiotics for comparison. This accounted for 84.0% of the extracted prescriptions. Prescriptions for the remaining short-term antibiotics prescribed for >28 days were excluded. This exclusion of these prescriptions together with those for more than 6 months’ supply excluded 1.49% of the prescriptions.

Dosing regimens were inferred from the specific antibiotic and quantity supplied (Supplementary Table S1). Prescriptions for 56 oxytetracycline tablets or more in the long-term analysis were included as it was decided that there were no other likely indications. All analyses were undertaken in Stata statistical software version 14.0. The use of CHIA data within this study was approved by the Care and Health Information Governance Group.

### Patient and public involvement

A patient and public involvement representative provided input into this work and commented that the work was important, particularly in terms of ‘informing future antibiotic prescribing guidelines in adolescents in order to weigh up the risk associated with prescribing prolonged courses’.

## RESULTS

**Figure 1.**
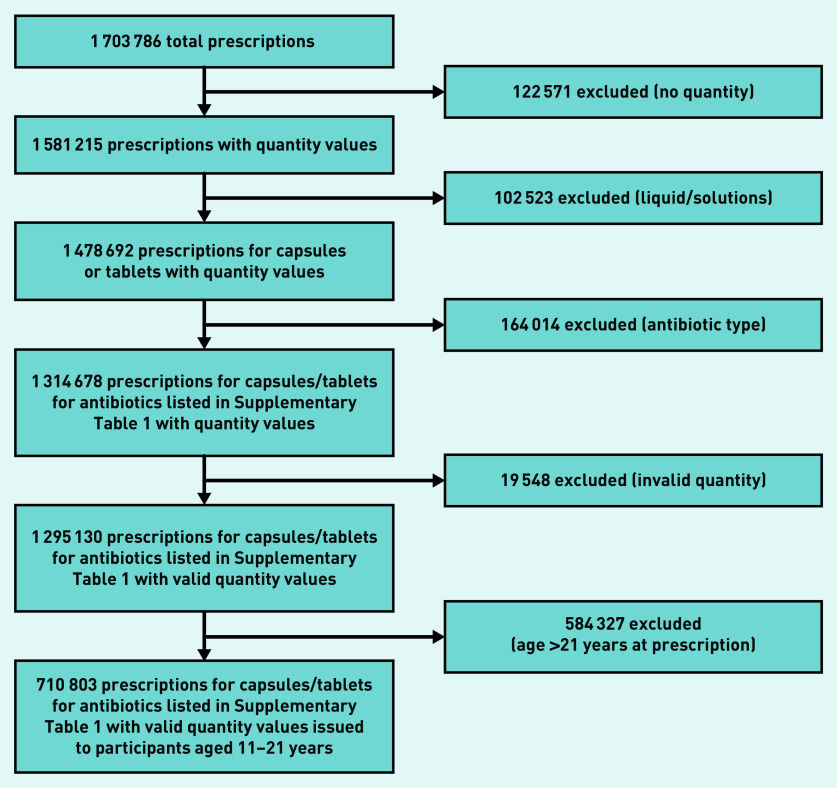
*Data selection and exclusion process.*

There were 320 722 participants who attended one of the included GP surgeries at least once during adolescence. A total of 1 703 786 antibiotic prescriptions issued between 1 January 1998 and 9 August 2017 at age 11 onwards were identified. There were 122 571 prescriptions that did not contain unit values for the quantity of medication issued. There were 102 523 liquid/solution prescriptions further excluded (as the duration could not accurately be inferred from quantity supplied). The analysis was limited to the 11 antibiotics listed in Supplementary Table S1 (excluding 164 014 prescriptions). It was necessary to further exclude 19 548 prescriptions with suspected invalid quantities >28 days for short-term antibiotics and >168 days for long-term antibiotics. The analysis was further restricted to prescriptions issued for patients between ages 11 and 21 (584 327 exclusions). The following analysis includes a total of 710 803 prescriptions. The data selection/exclusion process is depicted in Figure 1.

Table 1 lists a breakdown of the prescriptions included in the analysis by individual antibiotic. There were 157 056 patients (48.97%) who had prescriptions issued between the ages of 11 and 21, and the median age at first prescription was 14 years. In total, 11.01% of the CHIA cohort described here were prescribed at least one course of long-term antibiotics during the entire study period. The median follow-up was 10 years. Of the participants, 50.29% were female and 49.71% male. Between 185 and 264 GP surgeries contributed data in each year. The denominator of participants was lower towards the end of the study because of the cohort selection (to enable long-term follow-up data to be analysed for future work). Supplementary Table S2 details deprivation by decile of the cohort.

**Table 1.** Proportions of total prescriptions for individual antibiotics (710 803 prescriptions) at age 11–21 years

	**Prescriptions, %**	**Prescription days, %**
**Total short-term antibiotics**	**73.07**	**27.52**
Acute amoxicillin	19.78	7.18
Acute penicillin V	15.28	4.48
Acute flucloxacillin	13.93	5.70
Acute trimethoprim	8.57	2.30
Acute erythromycin	7.56	4.13
Acute co-amoxiclav	4.08	1.77
Acute doxycycline	2.22	1.38
Acute nitrofurantoin	1.65	0.57

**Total long-term antibiotics**	**26.93**	**72.48**
Long-term lymecycline	7.77	24.16
Long-term oxytetracycline	7.63	14.94
Long-term minocycline	6.07	18.99
Long-term erythromycin	2.43	5.31
Long-term doxycycline	1.92	5.84
Long-term trimethoprim	0.67	2.16
Long-term nitrofurantoin	0.44	1.08

**Figure 2.**
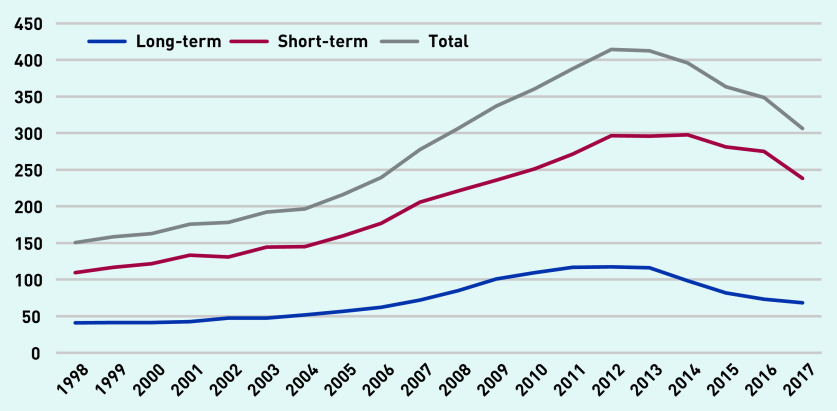
*Total antibiotic prescription items per 1000 person-years (at age 11–21 years).*

Figure 2 shows the rate of antibiotic prescriptions per 1000 person-years in the cohort from 1998 to 2017. The rate of total antibiotic prescribing increased from 150 per 1000 person-years in 1998 to just under 409 in 2012 and declined to around 306 in 2017. There were a total of 191 443 prescriptions (26.93%) for long-term antibiotics (≥28 days and ≤6 months in duration).

**Figure 3.**
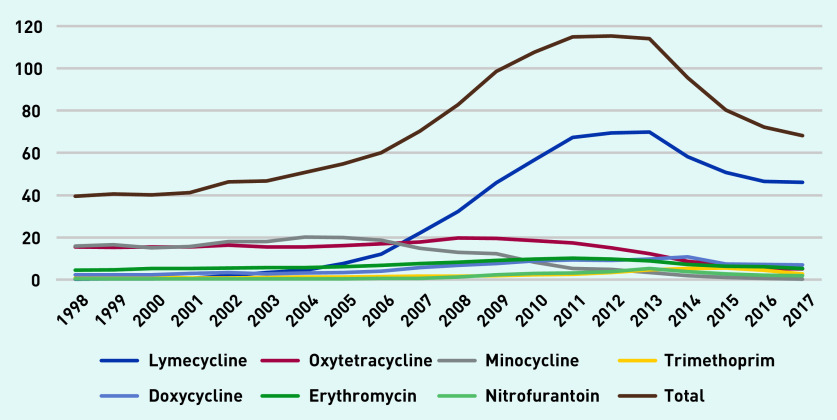
*Long-term antibiotic prescriptions per 1000 person-years (at age 11–21 years).*

Figure 3 depicts long-term antibiotic prescription rates per 1000 person-years. Tetracyclines were often prescribed and minocycline and oxytetracycline were the most prescribed long-term antibiotics up until 2007, after which lymecycline was the most commonly prescribed. Lymecycline prescribing increased to a peak of just over 69 prescriptions per 1000 person-years in 2013, declining to 46 in 2017.

**Figure 4.**
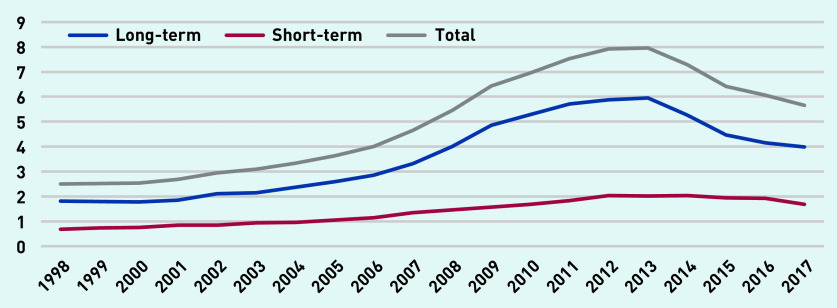
*Long-term, short-term, and total antibiotic prescription days per person-year (at age 11–21 years).*

Figure 4 depicts long-term, short-term, and total prescription days per person-year. Total long-term antibiotic prescribing peaked in 2013 at just under 6 days per person-year and declined to around 4 days in 2017. Total short-term antibiotic prescription days peaked at just over 2 days per person-year in 2014 and declined to 1.6 days in 2017. Combining total prescription days and total prescriptions data yielded an average duration of 6.7 days for short-term prescriptions and 49 days for long-term prescriptions.

**Figure 5.**
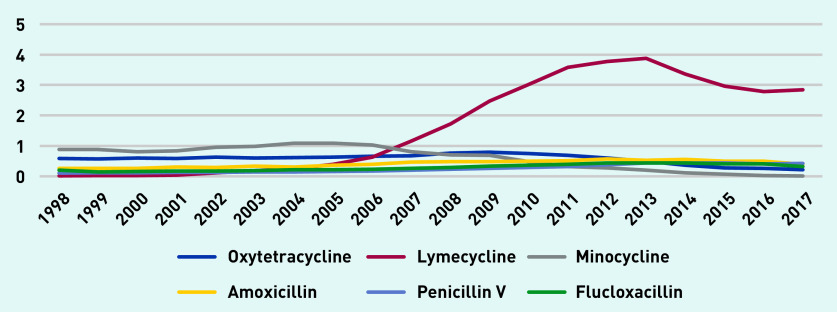
*Antibiotic prescription days per person-year for commonly prescribed antibiotics (long-term lymecycline, oxytetracycline, and minocycline versus short-term amoxicillin, flucloxacillin, and penicillin V) (at age 11–21 years).*

Figure 5 shows antibiotic prescription days per person-year for the most commonly prescribed long-term antibiotics (lymecycline, oxytetracycline, and minocycline) and also the most commonly prescribed short-term antibiotics (amoxicillin, flucloxacillin, and penicillin V). In 1998, minocycline prescriptions totalled 0.89 days per person-year compared with 0.19 for amoxicillin. Lymecycline prescriptions peaked at 3.87 days per person-year in 2013 and declined to 2.84 in 2017 compared with 0.43 for penicillin V in the same year.

## DISCUSSION

### Summary

This large community-based study demonstrates high rates of antibiotic exposure related to long-term antibiotic prescribing for adolescents and young adults primarily in the second decade of life. Long-term antibiotic prescribing accounted for more than two-thirds (72.48%) of total antibiotic exposure days. A significant proportion of the cohort (11.01%) were prescribed at least one long-term antibiotic prescription, most commonly lymecycline, which is commonly used in the treatment of acne.

### Strengths and limitations

The frequency of antibiotic dosing was inferred from the quantity supplied and this could affect the accuracy of the results, although dosing regimens for the most commonly prescribed antibiotics including lymecycline and amoxicillin are likely to be correctly inferred. Medication adherence was unknown for both acute and long-term antibiotic prescriptions. The study did not attempt to determine the indication for the antibiotic prescriptions and prescriptions that were not linked with Read codes were included. There was a significant number of prescriptions with missing quantity data (7.19%) and liquid preparations were not included in the analysis. The proportion of scripts for each individual antibiotic was similar in the missing data group and those included in the analysis.

The CHIA encompasses extensive coverage of approximately 75% of the resident Hampshire population at the time of data extraction. Although not all local practices participate, those that are missing are dispersed across the catchment area, with varied rural/urban classification, socioeconomic deprivation, and patient composition. The authors are not aware of any systematic differences to those practices whose data are present,^19^ and, although Hampshire has lower antibiotic prescribing rates than the national average,^20^ the CHIA prescription rates are comparable with large national databases such as THIN (discussed below). Further research using other healthcare databases could help confirm the current findings.

### Comparison with existing literature

In this analysis of routinely collected UK general practice data in Hampshire, relatively similar prescribing rates of individual antibiotics were found to those obtained by Dolk *et al* using the THIN database including similar total antibiotic prescription rates (413 per 1000 compared with 580 per 1000 person-years in patients aged under 19 in 2013).^9^ A study using the Clinical Practice Research Datalink (CPRD) database determined there were 489 prescriptions per 1000 person-years in 2017 (without age restrictions)^21^ compared with 306 prescriptions in the currently presented cohort. The difference could be because of higher proportions of prescriptions for older adults and very young patients included in the CPRD data. UK national data yielded an equivalent of 5 antibiotic days per person-year in 2017 for the entire population compared with 5.66 in this cohort in 2017.^22^ National data confirm an overall increase in antibiotic consumption in primary care, which peaked in 2014 and could have been driven by an increase in antimicrobial resistance.^22^ National data have also confirmed a fall since the 2014 peak because of the introduction of national targets implemented for general practice with financial incentives, education tools for prescribers, and patient leaflets.^22^

In the present study, 11.01% of participants aged 11–21 were prescribed at least one course of long-term antibiotics. Tetracyclines were the most commonly prescribed long-term antibiotics and this trend was observed in another study using the CPRD database.^16^ Tetracyclines comprised 7.9% of prescriptions in those aged under 19 years and 14.0% in those aged 19–65 in the Dolk *et al*^9^ study compared with 19.4% (for all doxycycline, lymecycline, oxytetracycline, and minocycline prescriptions) in 2014 in this study. In national data, lymecycline prescribing comprised 12.37% of all general practice antibiotic prescription days per person-year in 2017.^22^

Lower rates of lymecycline prescribing were found using CPRD data,^16^ where prescriptions were extracted for patients who consulted with any acne Read codes (11.8 items per 1000 person-years in 2013), but prescriptions may happen without a consultation code being recorded. As the current study did not link prescriptions with Read codes or consultations, it probably includes a more complete assessment of relevant prescriptions. There were relatively low rates of prescribing of antibiotics for urinary tract infections in this cohort as indicated in Table 1.

The number of antibiotic prescription days per person-year for long-term antibiotics was much greater than for commonly prescribed acute antibiotics and reflects considerable antibiotic exposure. In 2017, long-term antibiotics were prescribed just under 4 days per person-year compared with 1.7 days for acute prescriptions. These data suggest that long-term antibiotics (comprising around 70.38% of the total exposure in this cohort in 2017) and particularly lymecycline prescriptions comprise a major burden of antibiotic exposure in adolescents and young adults in the UK. It is feasible that this level of exposure could contribute significantly to AMR. A review and meta-analysis of faecal carriage of antibiotic-resistant *Escherichia coli* in asymptomatic young people found that resistance to many primary care-prescribed antibiotics is common and tetracycline resistance rates were high.^23^ Furthermore, healthy children carry bacteria resistant to antibiotics to which they are not usually exposed and resistance to tetracyclines could be acquired from family members or other children.^24^

Lymecycline is primarily prescribed for acne in adolescents and young adults, and recent National Institute for Health, and Care Excellence guidance for acne has published recommendations that include non-antibiotic treatment choices for acne of any severity.^12^ Recent research indicated that one in four patients with acne are prescribed oral antibiotics during a new acne consultation.^16^ Action is required to increase use of, and adherence to, alternative and effective topical therapies and for timely review of response to antibiotic therapy.

Treatment of acne with antibiotics is associated with an increased risk of common infections.^14^^–^15 In a retrospective cohort study of 84 977 individuals with acne treated with a topical antibiotic, oral antibiotic, or both, the odds ratio of developing an upper respiratory tract infection diagnosed by a GP was 2.15 times higher compared with patients not treated with antibiotics (*P*<0.001).^14^ These findings were supported by a subsequent cross-sectional study, in which self-reported pharyngitis was nearly twice as common in those exposed to oral antibiotics (66.7% compared with 36.2%).^15^ A potential explanation for this finding may be the depletion of natural oral flora supported by the finding that recolonisation therapy with a streptococcal spray has been shown to protect against recurrent infections.^25^

Antibiotics have been shown to have profound and sometimes persisting effects on the intestinal microbiota, characterised by diminished abundance of beneficial commensals and increased abundance of potentially detrimental microorganisms that can persist for years,^26^ and it is possible that long-term antibiotics may cause more profound and persistent changes. Long-term antibiotic use has been associated with a range of adverse outcomes including increased risk of colorectal adenoma,^27^ an increased risk of future cardiovascular events among older females at usual risk,^28^ and weight gain.^29^ Tetracyclines are also used as growth promoters in livestock to promote host lipid metabolism, energy harvest, and weight gain.^30^ Tetracycline usage during the fourth decade of life was, however, associated with reduced odds of obesity at enrolment to the Sister study.^29^ Other work using retrospective cohort data has suggested that long-term antibiotic use in healthy adolescents with acne was not associated with weight gain.^31^

### Implications for practice

Importantly, this study has shown that, in this cohort of adolescents and young adults, population exposure to long-term antibiotics is larger than that for acute antibiotics. Repeat use of long-duration antibiotics is associated with greater risk of antimicrobial resistance, and cross-resistance can occur where exposure to a specific antibiotic can induce resistance to other classes. Urgent action is needed by policymakers to curtail the use of long-term antibiotics, primarily lymecycline, for acne and promote the use of, and adherence to, alternative management strategies.
